# Bilateral total hip arthroplasty in a case with fibrous dysplasia: a case report

**DOI:** 10.1186/s13256-023-04084-9

**Published:** 2023-08-30

**Authors:** Alireza Moharrami, Peyman Mirghaderi, Mir Mansour Moazen-Jamshidi, Nima Hoseini Zare, Sadula Sharifpour, S. M. Javad Mortazavi

**Affiliations:** 1 Joint Reconstruction Research Center, Tehran University of Medical Sciences, Imam Khomeini Hospital Complex, End of Keshavarz Blvd, Tehran, Iran; 2 Surgical Research Society (SRS), Students’ Scientific Research Center, Tehran University of Medical Sciences, Tehran, Iran

**Keywords:** Arthroplasty, Case report, Fibrous dysplasia of bone, Hip

## Abstract

**Background:**

Fibrous dysplasia (FD) is a benign neoplasm with a broad spectrum of presentations. The treatment of FD in the hip region is controversial among orthopedic surgeons. Several treatment options exist, including curettage and grafting, valgus osteotomy, medial displacement osteotomy, and so on. Performing total hip arthroplasty (THA) on these patients and their subsequent outcome is still in infancy.

**Case presentation:**

The patient is a 32-year-old white female with bilateral proximal femur FD who underwent bilateral THA with long stem implants. A year following surgery, she had no complications and had satisfactory radiological, pain, and functional outcomes.

**Conclusion:**

A bilateral THA with a long stem prosthesis showed promising results when performed following appropriate curettage of the proximal bone in an FD case. A cementless long stem could have enabled better diaphyseal fixation distal to the lesion site.

## Background

Fibrous dysplasia (FD) is a benign neoplasm with a broad spectrum of manifestations as a developmental anomaly [1]. This benign bone tumor comprises fibro-osseous tissue and accounts for approximately 5% of all benign bone tumors [1]. The disease can affect a single bone (monostotic) or multiple bones (polyostotic). Polyostotic tumors can be associated with endocrinopathies, including hyperthyroidism, hyperparathyroidism, precocious puberty, Cushing syndrome, and phosphate-wasting osteomalacia [1]. Polyostotic tumor involvement is associated with cafe au lait spots, known as McCune-Albright syndrome. However, monostotic tumors are ten times more prevalent than polyostotic tumors [1].

The proximal femur is the most frequently affected site, often accompanied by pathological fracture [2]. The best treatment options for FD in the proximal femur remain controversial [2]. The possible options are curettage and grafting, valgus osteotomy, and medial displacement osteotomy [2, 3].

In the case of recurrent FD, a total hip arthroplasty (THA) may be necessary as a last resort [4–6]. To our knowledge, only a few reports demonstrate outcome of THA in managing FD [7, 8]. The purpose of this study is to describe a case of bilateral proximal femur FD which was treated with bilateral THA.

## Case presentation

### Patient information

A 32-year-old white female patient (BMI = 24) visited our clinic with complaints of bilateral hip pain for the last 10 years. She had a history of limping since childhood and had undergone bilateral Salter osteotomy at three. She had no other medical history and did not use any medication.

### Clinical findings

On examination, she had a surgical scar on the lateral side of her left thigh. She had limitations in her right hip internal rotation and had a Trendelenburg gait. Her true lower limb length was 83 cm on the right and 84 cm on the left side, and her apparent lower limb length on the right and the left side was 87 and 90 cm, respectively. The American Society of Anesthesiologists (ASA) classified her as grade 1.

### Diagnostic assessment

**Fig. 1:**
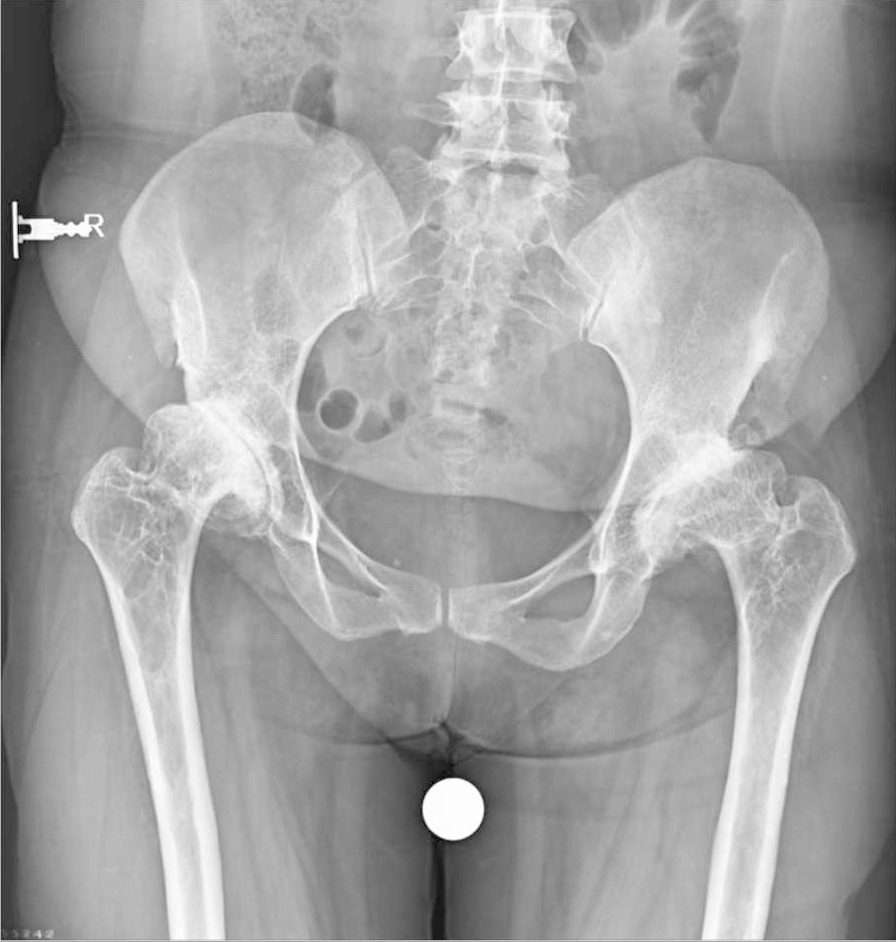
Plain hip radiograph of the patient

**Fig. 2:**
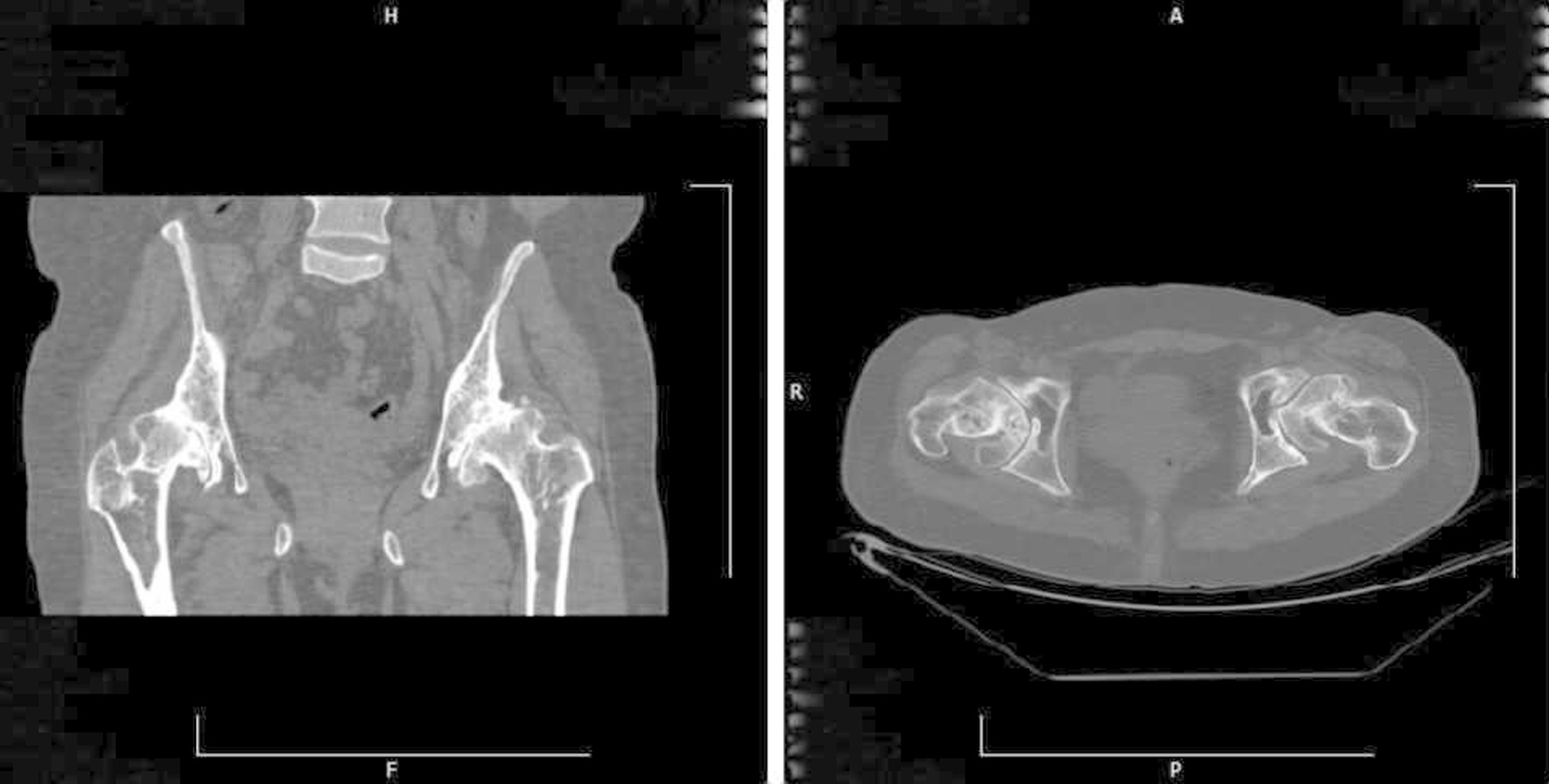
The pelvic Spiral CT scan in coronal (right) and axial (left) views

**Fig. 3:**
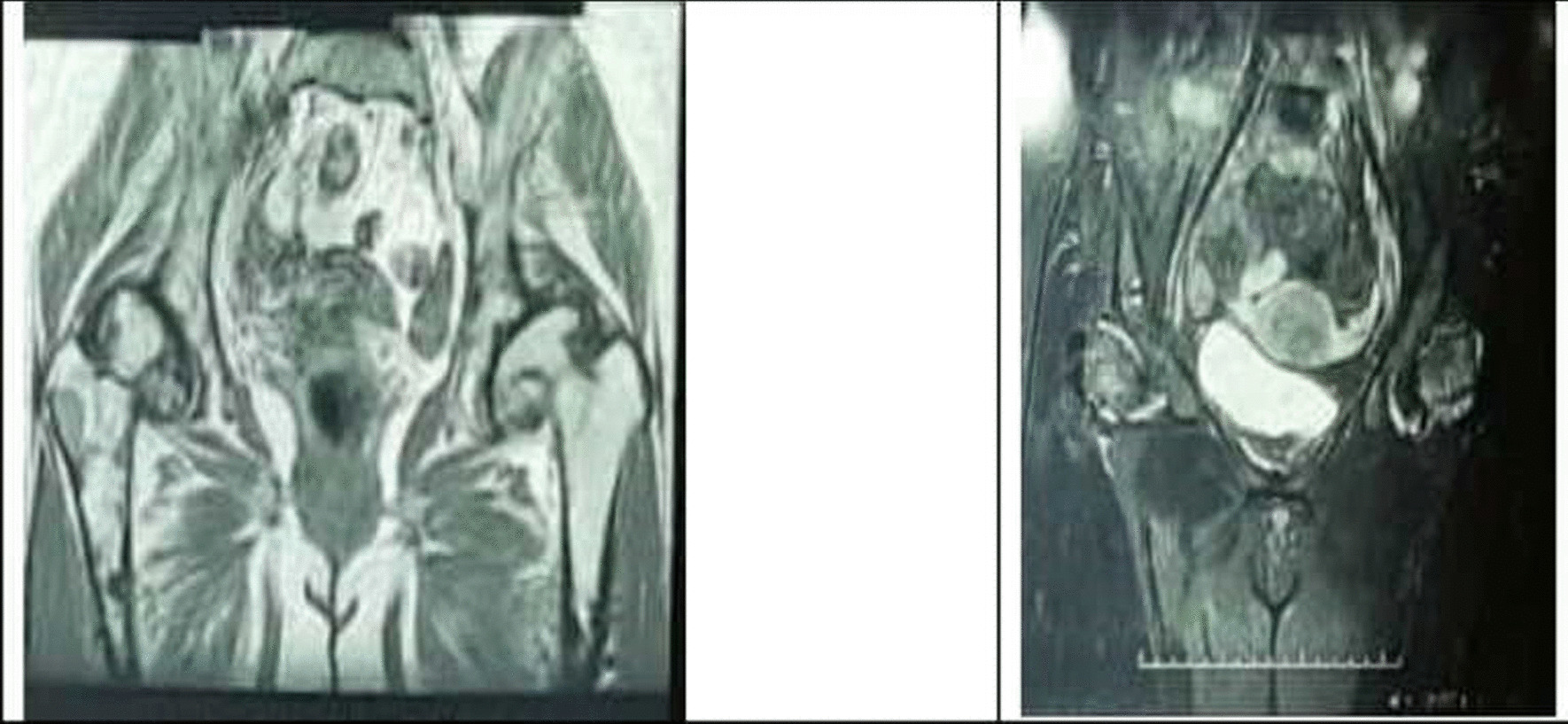
Pelvic MRI of the patient (T1: left, T2: right)

**Fig. 4:**
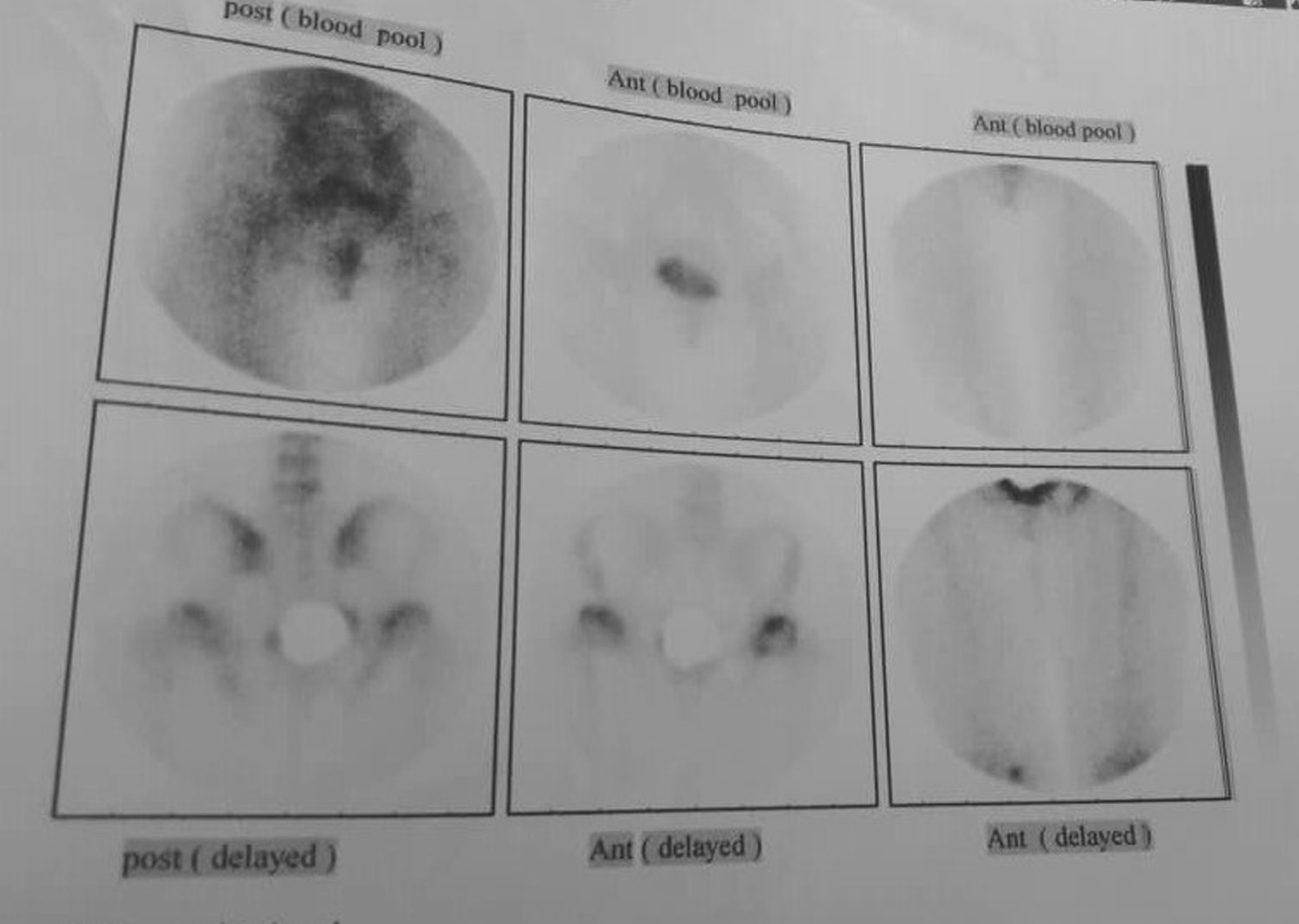
Bone scan of the patient that showed there is not any uptake

On a plain pelvic x-ray, there were ground glass lesions on both sides of the proximal femur region (Fig. 1). She had severe joint degeneration in both hips. The right and left hip acetabular index were 42.3 and 48.9, respectively. The lateral center edge angle was measured at 6° on the right and 15° on the left side. According to the CT scan, there were lytic lesions in the proximal femur on both sides, but there was no evidence of a pathologic fracture (Fig. 2). She had a type 2 Paprosky acetabular bone loss on the right side and a type 1 Paprosky acetabular bone defect on the left side [9]. The lesions in the proximal femur were characterized by a high signal on T1 and a low signal on T2 on magnetic resonance imaging (MRI) (Fig. 3). On bone scan imaging, mildly increased uptake was observed in the trochanteric and subtrochanteric regions of her right femur. No other abnormal uptake was detected (Fig. 4).

### Therapeutic intervention

**Fig. 5:**
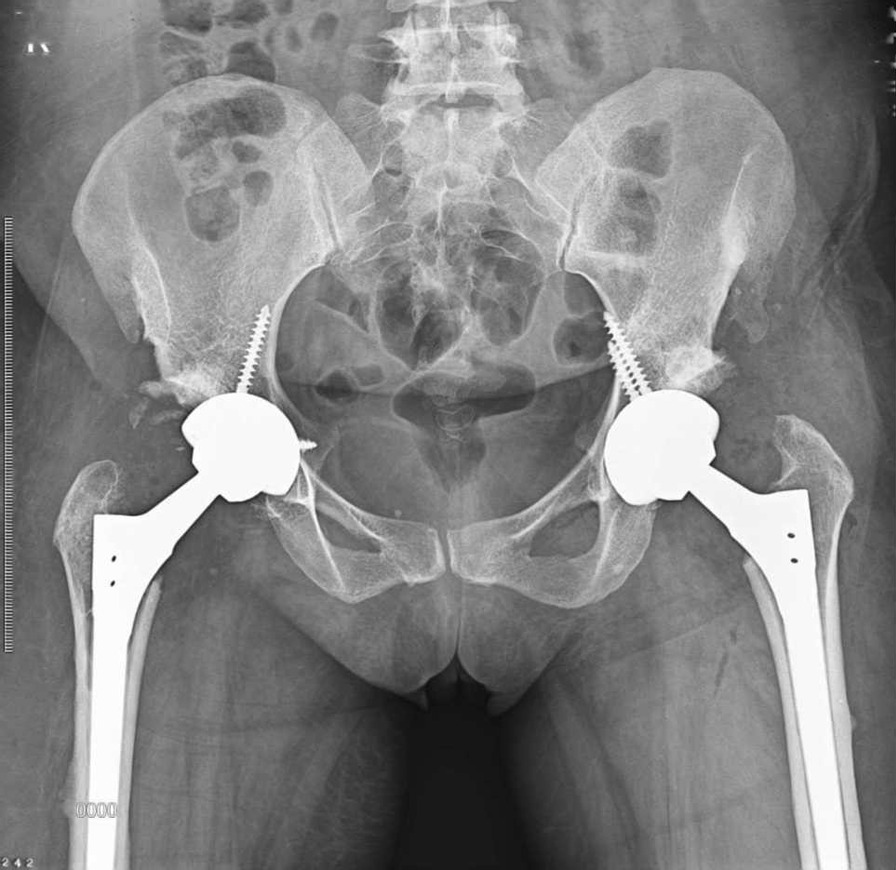
Plain hip x-ray of the patient after bilateral hip arthroplasty

After preoperative planning for implant size and radiological factors [10], simultaneous bilateral THA surgery was scheduled for her [11] (Fig. 5). In the operating room, the Smith-Peterson approach was selected. After a thorough curettage of the proximal femoral lesion, bone allograft was used. Finally, cementless THA was performed on both sides using a long-stem femoral component. Although the preoperative assessment indicated that this case was not a typical instance of FD, during the operation, the presence of FD was suspected. To confirm our suspicions, we sent a pathology sample to the laboratory for analysis. Results from the laboratory confirmed that the patient was indeed suffering from FD.

### Follow-up and outcomes

**Fig. 6:**
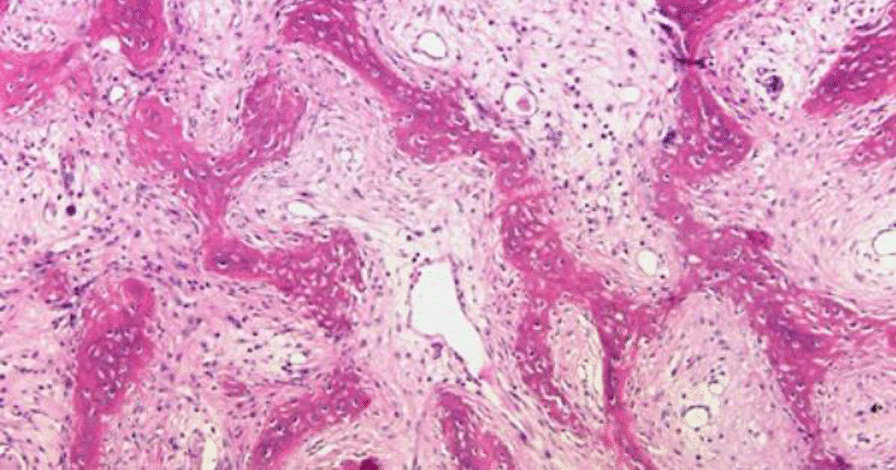
The high power field view of fibrous dysplasia pathology in our patient

**Fig. 7:**
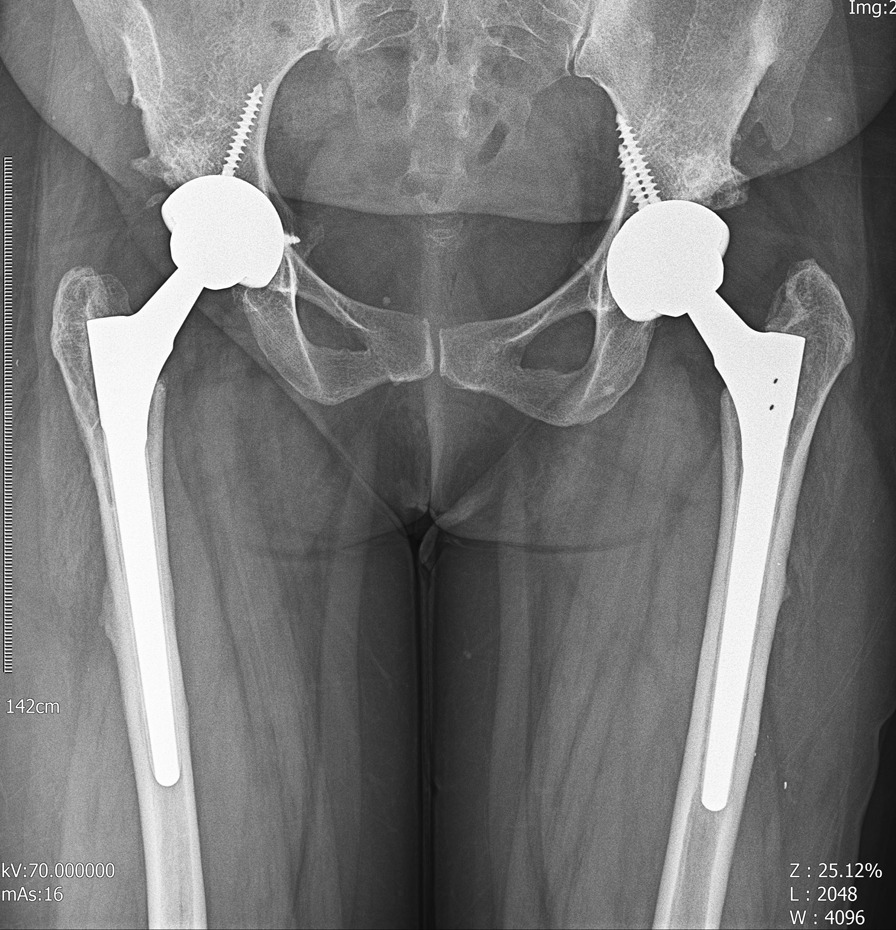
The last pelvic x-ray of our patients 12 months after the surgery

After the procedure, she experienced an uneventful recovery, and her pain subsided significantly on both sides. The pathological evaluation demonstrated fibrodysplasia by an expert musculoskeletal pathologist (Fig. 6). After a 12-month follow-up, the patient had acceptable radiological and functional results (Fig. 7). She reported a Harris Hip Score (HHS) of 26 preoperatively, improving to 81 a year after the arthroplasty.

## Discussion and conclusions

Patients with FD account for approximately 5% of all benign bone tumors. FD patients are likely to require THA at some point in their lifetime. However, limited information is available regarding the results and complications of this procedure [12].

Several treatment modalities have been used to treat proximal bone involvement. Curettage and bone grafting, with or without osteotomies, are among them [2, 3]. A curettage alone is unlikely to eliminate the tumor but can weaken the bones and leave them vulnerable to stress fractures. Furthermore, it is possible that grafting will resorb over time and be of no benefit [2, 13]. Also, it may be difficult to perform osteotomies in a weak polyostotic case, and you may require internal fixation such as intramedullary nails [3]. If the condition recurs, some may advocate a complete resection or even an amputation [14].

Garceau *et al.* studied ten hips with FD that underwent THA, with a mean follow-up of 6 years [15]. They showed that THA in FD patients is associated with low complication (Two transient sciatic nerve palsies) and revision rates (one hip) using cementless components and allograft. Their technique involved using uncemented conical fluted stems, which provided better distal diaphyseal fixation. They hypothesized that this type of prosthesis would improve the surgical outcome and have longer longevity [15]. Moreover, they suggest that the allograft may be useful for polyostotic FD patients with poor bone stock. Yao *et al.* [16] treated twelve patients with proximal femur fractures and hip joint osteoarthritis with 3D designing osteotomies for deformity correction and THA for hip osteoarthritis. By using preoperative 3D design, corrective osteotomy, and long cementless stems, they could precisely restore alignment, preserve bone stock, and achieve stem stability. As a result, we can expect long-term longevity and better functional results [16]. The study by Sierra *et al.* reported excellent pain relief and function in 11 patients with FD treated through THA before 2001 [12]. However, the higher surgical complication rate and the need for revision (7 hips) should be noted. In their study, 7 hips were implanted with cementless stems, and 5 hips were implanted with fully or proximally coated cementless stems. To bypass bone defects, they used cemented stems.

We represented a bilateral THA for treating FD in both proximal femurs using a cementless long stem through a Smith-Peterson approach. During the first year of follow-up, we found this technique acceptable and without complication, providing good clinical outcomes. No early loosening of the prostheses was observed. A cementless long stem could have enabled better diaphyseal fixation distal to the lesion site.

A limited number of studies have demonstrated that THA is an effective treatment option for proximal femur involvement of FD, particularly in cases of recurrence [8, 15, 16]. It may be necessary to use a cementless long-stem component for THAs in cases of proximal femur DF. In this way, Paprosky classification can help evaluate the acetabular bone loss and guide the choice of an appropriate prosthesis. The results of our study suggest that bilateral THA with cementless long-stem implants may be a viable treatment option in patients with bilateral proximal femur FD when performed after adequate curettage.

## Data Availability

Not applicable

## Abbreviations

FD: Fibrous dysplasia
CT: Computed tomography
MRI: Magnetic resonance imaging
THA: Total hip arthroplasty
